# Proteomic Insights into Childhood Obesity: A Systematic Review of Protein Biomarkers and Advances

**DOI:** 10.3390/ijms26178522

**Published:** 2025-09-02

**Authors:** Dominika Krakowczyk, Kamila Szeliga, Marcin Chyra, Monika Pietrowska, Tomasz Koszutski, Aneta Gawlik-Starzyk, Lidia Hyla-Klekot

**Affiliations:** 1 Saint John Paul II Upper Silesian Child Health Centre, Public Clinical Hospital no.6 of the Medical University of Silesia in Katowice, Medyków 16 Street, 40-752 Katowice, Poland; kszeliga@gczd.katowice.pl (K.S.); tkoszutski@gczd.katowice.pl (T.K.); agawlik@gczd.katowice.pl (A.G.-S.); chirurgia@gczd.katowice.pl (L.H.-K.); 2 Department of Pediatric Neurology, Independent Public Healthcare Centre—Municipal Hospital Complex, ul.W. Truchana 7, 41-500 Chorzow, Poland; marcin-chyra@wp.pl; 3 Maria Sklodowska-Curie National Research Institute of Oncology, 44-102 Gliwice, Poland; monika.pietrowska@gliwice.nio.gov.pl

**Keywords:** childhood obesity, proteomics, biomarkers

## Abstract

Childhood obesity has emerged as one of the most pressing public health challenges of the 21st century. Early-onset obesity is associated with an increased risk of developing numerous comorbidities later in life. Despite extensive research into its multifactorial etiology—including genetic, behavioral, environmental, and socioeconomic factors—the precise molecular mechanisms underlying the development and persistence of obesity in the pediatric population remain incompletely understood. Proteomics offers promising insights into these mechanisms. The application of proteomics in pediatric obesity research has grown, enabling the identification of proteins that reflect dynamic changes in metabolic and inflammatory pathways. This advancement allows clinicians to move beyond traditional anthropometric measurements toward personalized approaches with notification of early complications of obesity. A systematic search was conducted across PubMed, Scopus, and Web of Science for studies published between 2010 and 2025. Inclusion criteria: human studies, participants aged 0–18, proteomic analysis of obesity, and biomarkers. Data extraction and quality assessment followed standardized protocols. From 239 articles, 20 were included. Key dysregulated proteins include APOA1, CLU, and HP. LC-MS/MS was the predominant technique used. Some biomarkers were predictive for obesity complications in children. Proteomics holds clinical potential for early detection and personalized treatment of pediatric obesity. Standardized methodologies and longitudinal studies are needed for translation into clinical practice.

## 1. Introduction

Childhood obesity has emerged as one of the most important public health challenges of the 21st century. Its global prevalence has increased at an alarming rate, with the World Health Organization (WHO) estimating in 2020 that over 39 million children under the age of five were overweight or obese. Numbers are continuing to rise, particularly in low- and middle-income countries, and they have been increasing more rapidly in children than in adults [1]. Childhood obesity is a complex health issue arising from interactions among genetic factors and changing environmental influences—particularly modern dietary patterns—that together disturb the balance between calorie intake and energy burned [2,3]. This imbalance initiates a cascade of metabolic disorders, creating the conditions for various negative health consequences [4]. The early-onset form of obesity is associated with an increased risk of developing metabolic syndrome, hypertension, type 2 diabetes mellitus, chronic kidney disease, systemic inflammation, cardiovascular disease, and psychological comorbidities later in life [5,6,7,8,9,10].

Early diagnosis is essential in arranging well-coordinated, specialized, and multidisciplinary care, requiring active collaboration among healthcare providers, social services, and families. Preventing the worsening of obesity involves controlling food availability, creating a supportive and structured eating routine, and encouraging consistent, tailored, and supervised physical activity on a daily basis [11,12,13,14,15].

Despite extensive research into its multifactorial etiology, the precise molecular mechanisms that cause and maintain obesity in pediatric populations remain incompletely understood.

One of the greatest challenges in addressing childhood obesity is detecting the condition and its metabolic consequences early, often before serious health problems arise. Traditional anthropometric and biochemical assessments offer limited insights into the complex biological networks that drive the onset and progression of obesity and its comorbidities [16,17].

In modern medical diagnostics, multi-omics approaches have emerged as a novel and promising field. In our study, we focused specifically on proteomic techniques as a tool for identifying potential biomarkers of metabolic dysfunction, such as proteins, altered lipid profiles, inflammatory responses, and hormonal imbalances. (In Supplementary File S1, we present the foundational principles of proteomic techniques.) By identifying these biomarkers in obese children or those exhibiting subclinical metabolic dysfunction, proteomic analysis may facilitate the early identification of at-risk individuals and enable timely interventions to prevent the onset of obesity-related conditions [18].

Consequently, there is a growing interest in identifying reliable biomarkers that can facilitate early diagnosis, risk stratification, and targeted intervention. There is increasing recognition that personalized treatment strategies are needed in order to address obesity effectively. Proteomic studies can help in the creation of personalized obesity management strategies by identifying distinct proteomic signatures in different obesity subtypes. For example, the response to dietary changes, physical activity, bariatric surgery, changes in the growing organism, or pharmacological treatments could be predicted based on protein expression profiles [13,19,20,21,22,23,24,25,26]. In our review, we have focused on studies on biomarkers in the pediatric population where proteomic studies were used.

This review aims to synthesize the current state of knowledge regarding proteomic biomarkers in childhood obesity, highlighting key methodological approaches, the protein signatures identified, and their relevance to clinical phenotypes. These integrated models will also help clarify the interactions between genetic predisposition and environmental factors, providing a more holistic understanding of childhood obesity.

## 2. Materials and Methods

This systematic review was conducted in accordance with Preferred Reporting Items for Systematic Reviews and Meta-Analyses (PRISMA) guidelines. A structured literature search was performed across three major databases and registers: PubMed, Scopus, and Web of Science, and through websites and citation searching, covering studies published between January 2010 and April 2025.

**Figure 1:**
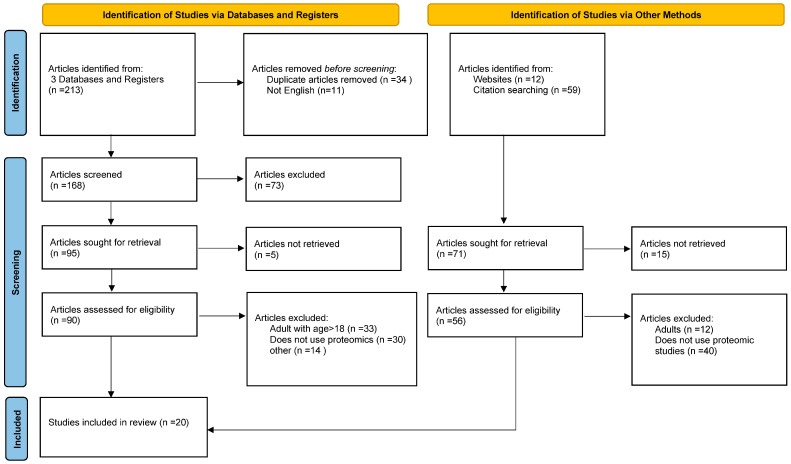
PRISMA flowchart for study selection process.

The detailed search strategy is available in Supplementary File S2. The study selection process is illustrated in the PRISMA flowchart (Figure 1).

Inclusion criteria:

Studies involving human participants aged 0–18 years.

Participants classified as overweight or obese.

Use of proteomic analysis to identify or assess biomarkers.

Original peer-reviewed articles, including cohort, case-control, cross-sectional, or interventional designs.

Studies published in English.

Exclusion criteria:

Animal or in vitro studies.

Studies involving only breastfeeding infants or incorrect age group.

Papers using general protein measurements without applying a proteomic approach.

Reviews, editorials, and conference abstracts.

Study selection and data extraction:

Two independent reviewers [D.K.&K.Sz] screened all titles and abstracts. Full-text articles were retrieved for potentially eligible studies and assessed for final inclusion. Any disagreements were resolved through discussion or consultation with a third reviewer.

A standardized data charting form was developed and pilot-tested. For each included study, we extracted the following information:

Study characteristics (authors; year of publication).

Participant demographics (age, sex, BMI, and comorbidities).

Sample type and proteomic platform used.

Identified biomarkers and proteins.

Main outcomes and clinical correlations.

Supplementary File S2—Proteomic article search algorithm.

Although the systematic search informed the structure of this review, not all included studies were subjected to quantitative synthesis. Instead, the extracted data were used to support a narrative synthesis of the current state of proteomic biomarker discovery in pediatric obesity. Studies were categorized according to the types of behaviors they examined, and for each category, the settings, populations, and study designs were summarized, along with the measurement approaches and overarching findings. The systematic review framework was applied solely to describe and discuss the methodological aspects; these studies were therefore not included in our primary Table 1.

## 3. Results

The literature search, including records identified via databases and registers [“Articles screened (*n* = 168)”] and those identified via other methods [“Articles sought for retrieval (*n* = 71)”], has given rise to a total of 239 studies. Following the screening of titles and abstracts, 146 full-text articles were assessed for eligibility, and 20 studies met the inclusion criteria. The study selection process is presented in Figure 1.

Reasons for exclusion included participants not being the correct age, studies that involved proteins but did not constitute proteomics works, exclusively breastfeeding-related studies, animal studies, and incorrect study designs. All included studies are summarized in Table 1.

We identified more than 54 proteomic biomarkers or candidates/emerging biomarkers for childhood obesity, summarized key methodological approaches, detected protein signatures, and presented their functions. These are detailed in Table 2

Identifying biomarkers for early-onset obesity is crucial in the timely intervention and prevention of associated metabolic disorders. Recent research utilizing proteomic analyses has uncovered several potential biomarkers in children; we have chosen those that show the strongest correlation and are the most often repeated:

- APOA1 (Apolipoprotein-A1): Studies have shown a decrease in various isoforms of APOA1 in obese children compared to lean controls. This reduction is more pronounced in insulin-resistant individuals and may be partially reversed with weight loss. APOA1 particles can increase cholesterol efflux from cells and risk for cardiovascular disease [18,20,21,27,28,40,41,46,47,48].
- CLU (Clusterin; Apolipoprotein J): This is involved in lipid transport, apoptosis regulation, and protection against oxidative stress. Reduced levels of clusterin have been observed in obese pediatric populations, suggesting a compromised capacity to manage cellular stress and lipid transport. This protein is known to accumulate in the artery wall during the development of atherosclerosis and has been detected in the infarcted heart during myocardial infarction. Given its multifaceted role, alterations in clusterin levels may contribute to the pathophysiology of obesity and its related complications [30,31,39,49,50,51].
- APOE (Apolipoprotein E): This is involved in lipid transport and clearance [28].
- HP (Haptoglobin): An increase in low-molecular-weight isoforms of haptoglobin (as well as a decrease in high isoforms) has been observed in obese children, especially those with insulin resistance. These elevated levels correlate positively with inflammatory markers such as interleukin-6 and NAMPT/visfatin. It is also an acute-phase/inflammation marker [6,18,28,33,39,49,52,53,54].
- CFB (Complement Factor B), CFH (Complement Factor H), and CFI (Complement Factor I): These proteins are integral components of the immune system’s complement pathway. Elevated levels have been associated with an increased BMI, suggesting a link between obesity and immune system activation [8,9,20,22,28,31,32,33,39,40,43,55,56,57].
- VDBP (Vitamin D Binding Protein): This protein transports vitamin D metabolites in circulation. Lower concentrations in obese children may reflect alterations in vitamin D metabolism associated with obesity. Upregulation was paralleled by the adiponectin-interactive protein DsbA-L, suggesting that the VD regulation of adiponectin involves post-transcriptional events. Some pediatric studies link higher VDBP levels with higher HOMA-IR or impaired glucose metabolism, suggesting that VDBP may be involved in the vitamin D–insulin axis. Higher or altered VDBP levels in obese children compared to lean ones are often correlated with insulin resistance or inflammatory markers. Using a proteomic approach, multimeric adiponectin has been identified as a key plasma protein that links VDD with pediatric obesity [8,32,36,43,49,58].
- ADIPOQ (Adiponectin): This is a clinically relevant protein biomarker that reflects metabolic health in children with obesity. Its HMW form is especially significant in insulin sensitivity and is often reduced in obese, insulin-resistant, or vitamin D-deficient children [32].
- TTR (Hormonal Transthyretin): This transports thyroxine and retinol-binding protein. Decreased levels in obese children suggest potential impacts on thyroid hormone and vitamin A transport [32,49,59].
- PEDF (Pigment Epithelium-Derived Factor): Secreted by adipose tissue, this can induce insulin resistance and inflammatory signaling [7,31,48,57,60,61].
- RBP4 (Retinol-Binding Protein 4): This protein contributes to insulin resistance, vascular inflammation, endothelial dysfunction, and atherosclerosis. It also influences adipocyte differentiation and inflammation [10,20,31,57,62,63].
- PON1 (Paraoxonase 1): This plays a key antioxidant, anti-inflammatory, and detoxifying role. Alterations in its activity—often due to genetic polymorphisms—are associated with a broad spectrum of diseases, particularly those involving oxidative stress, inflammation, lipid dysregulation, and toxin exposure [5,58,64,65,66,67,68].
- ALDH (Aldehyde dehydrogenase): This is a family of enzymes that detoxify aldehydes (through lipid peroxidation, alcohol metabolism, etc.), contributing to oxidative stress control. ALDH isoforms are often upregulated in obese children, particularly in those with metabolic syndrome or fatty liver disease. This suggests a compensatory response to oxidative stress and lipid peroxidation associated with excess adiposity. It has been identified in plasma and liver tissue proteomics in obesity-related NAFLD and may serve as a marker of oxidative stress load or liver dysfunction in early obesity-related organ damage [27,28,69,70].
- ALB (Albumin): This is a major plasma protein; it maintains oncotic pressure and transports hormones, fatty acids, and drugs. In pediatric obesity, total albumin levels may remain normal, but post-translational modifications (e.g., glycation; oxidation) increase. Lower “functional” albumin may reflect oxidative stress or systemic inflammation. It is identified as differentially abundant or modified in obese vs. lean children in serum proteomic panels. Changes in albumin may reflect subclinical inflammation, oxidative damage, or early renal impairment in obesity [20,71,72].
- CD38 (Cluster of differentiation 38): This is a transmembrane enzyme that breaks down NAD^+^, a key cofactor in cellular metabolism. CD38 is heavily involved in diet-induced obesity and is the protein that shows the most consistent association with cardiometabolic and brain health. Its receptor is involved in immune cell activation, calcium signaling, and NAD^+^ metabolism. In pediatric obesity, CD38 levels have been found to be lower in metabolically healthy obese (MHO) children compared to metabolically unhealthy obese (MUO) ones. This suggests a potential protective role: lower CD38 may reflect reduced inflammation and a better metabolic profile. It is also linked to brain–immune signaling and blood–brain barrier integrity [40,73].
- LAIR2 (Leukocyte-Associated Immunoglobulin-Like Receptor 2) collagen-binding protein: This is a soluble immune modulator that inhibits immune cell overactivation by interfering with LAIR1. In pediatric obesity, it is lower in MHO children than in MUO children. It may reflect reduced chronic low-grade inflammation, making it a potential marker of a healthier immune–inflammatory status in obesity [40].
- MANF (Mesencephalic Astrocyte-derived Neurotrophic Factor): This is a neurotrophic factor that supports neuron survival and maintains endoplasmic reticulum (ER) function and cellular stress responses. It is reduced in metabolically healthy obese compared to non-metabolically healthy obese children. Its decrease in healthier obese children might suggest lower ER stress or better neural and metabolic balance, highlighting links between metabolic health and brain function [40].
- NRP2 (Neuropilin-2): This is a co-receptor for VEGF isoforms and plays a role in neurological diseases in obese children. It is involved in angiogenesis, neurodevelopment, and immune cell migration. In pediatric obesity, it is lower in MHO children. This suggests that there is improved vascular and neuroimmune regulation in metabolically healthier obesity phenotypes [40].
- HSP90AA1 (Heat Shock Protein beta 1 A, PCYOX1, and HSP90AA1): These are small heat shock proteins involved in cell protection, cytoskeletal stability, and anti-apoptotic functions. They may be upregulated as a cellular defense mechanism against metabolic or oxidative stress. Elevated levels may correlate with insulin resistance or inflammatory stress. They have been detected to be upregulated in obese children with insulin resistance in targeted proteomics studies. They represent a potential early marker of cellular stress and insulin resistance development, predictive for liver steatosis [20,44].
- PDIA3 (Protein Disulfide-isomerase A3): This is an ER-resident protein that assists in protein folding and redox regulation and is often upregulated in obese individuals’ children, indicating ER stress and misfolded protein response, which are common in insulin resistance and obesity. It has been identified in both the liver and plasma proteomes of obese pediatric patients, often in those with NAFLD. It may reflect ER dysfunction and metabolic inflammation, possibly preceding overt comorbidities [20,28,48,58,72].
- Plasma lyso-PEs, especially 16:0 and 22:6 species: Lyso-PEs are bioactive lipid intermediates, formed via the partial hydrolysis of phosphatidylethanolamines, typically by phospholipase A2. They influence membrane fluidity, cell signaling, and inflammation. A pattern of Lyso-PE 16:0 (palmitoyl-LysoPE) ↓ decreasing and Lyso-PE 22:6 (docosahexaenoyl-LysoPE) ↑ increasing was observed in overweight vs. normal-weight children even before classical lipid profile abnormalities. These changes may reflect early alterations in lipid metabolism, cellular stress, or low-grade inflammation and may indicate subclinical shifts in lipid signaling or mitochondrial lipid remodeling [20,41,61,62,74,75].
- IGFBP1 (Insulin-Like Growth Factor Binding Protein 1): This binds to IGF1, regulating its availability for tissues. It is inversely regulated by insulin—a high level of insulin suppresses IGFBP1 production. IGFBP1 is consistently found to be reduced in obese and insulin-resistant children. This reflects hyperinsulinemia, even before glucose tolerance declines. IGFBP1 (and IGFBP2) are often measured in proteomic studies via multiplex immunoassays, SOMAscan, or targeted mass spectrometry. These proteins are among the most reliable early indicators of metabolic dysfunction, especially when combined with inflammatory and lipid-related markers [55,76,77,78,79].
- CRP (C-Reactive Protein): A well-known marker of inflammation, CRP levels correlate strongly with BMI in obese individuals, indicating chronic low-grade inflammation associated with obesity [20,22,27,28,43].
- Calprotectin Complex (S100-A8/S100-A9): This complex is involved in inflammatory responses and has been found at higher concentrations in individuals with obesity, reflecting an ongoing inflammatory state [43,47].
- S100 Proteins (S100-A8 and S100-A9): These calcium-binding proteins form the calprotectin complex, which is involved in inflammatory processes. Their elevated expression in obese individuals further highlights the inflammatory component of obesity [80].
- PTX3 (Pentraxin 3) is a mediator of subclinical inflammation in atherosclerosis, which could serve as a sensitive early biomarker for cardiovascular risk in children, signaling inflammation before overt disease manifests [81,82].
- NEBL (Nebulette): This may reflect subclinical cardiovascular remodeling or cardiac stress in pediatric obesity [43].
- Galectin 3BP (Galectin-3 binding protein): This is a novel marker of obesity and metabolic syndrome [35].
- SEM4A, PSB3, DDC, C1RL1, T132A (uncharacterized), pyruvate carboxylase, and C1-esterase inhibitor: These proteins implicate inflammation, metabolic stress, proteostasis dysregulation, and immune activation in the early pathogenesis of diabetic kidney disease. They could be used as markers in early risk stratification and targeted interventions in obese youth-onset T2D [39].
- RAS-GTPase: This is involved in insulin signaling, activating the PI3K/Akt and MAPK pathways. Its dysregulation leads to insulin resistance. eIF4E Eukaryotic translation initiation factor 4E leads to translation initiation and metabolic stress [29].
- PKC-η (Protein kinase C-eta): This plays a role in inflammation signaling and activates inflammatory cytokine expression; it is overactive in obesity and contributes to IR (insulin resistance) [35,83].
- SSP1 (Osteopontin): This is a known bone remodeling and inflammatory marker. It indicates that in obese adolescents, inflammatory/integrity pathways play a key role in bone development and density changes [45].
- MFAP5 (Microfibrillar-associated protein 5): This is an extracellular matrix protein implicated in bone strength, used to identify youth at risk for poor bone accrual. It is particularly important in the context of excess adiposity [45].
- p38 MAPK (Mitogen-activated protein kinase): This is involved in inflammatory signaling (e.g., TNF-α; IL-6), insulin signaling modulation, adipocyte differentiation and lipolysis, and muscle and liver glucose homeostasis [29].
- AHSG (Fetuin A): Children with obesity or overweight consistently show elevated serum and salivary fetuin-A levels. Increased fetuin-A correlates with increased BMI, waist circumference, HOMA-IR (insulin resistance index), and triglyceride levels. Weight loss or physical activity programs can lower fetuin-A levels, reflecting improved insulin sensitivity and metabolic health [63].
- MSR1 (Macrophage scavenger receptor type I): Membrane glycoproteins are implicated in the pathological deposition of cholesterol in the arterial wall. This protein plays a role in clearing infectious agents and toxic molecules in pro- and anti-inflammatory responses and cardiometabolic and brain health [80].
- STAT3: Proteomic/phosphoproteomic studies in children are still emerging. However, STAT3′s downstream involvement in energy homeostasis, inflammation, and hypothalamic regulation suggest that it is an important therapeutic and mechanistic target in pediatric metabolic research [41].
- PDK1 (Phosphoinositide-Dependent Kinase 1): PDK1 is a central kinase downstream of PI3K, responsible for the phosphorylation and activation of Akt/PKB and other AGC kinases—key players in insulin, growth factor, and metabolic signaling. Though direct evidence in pediatric obesity is currently limited, PDK1′s role in insulin pathway functionality is well established. Future proteomic or phosphoproteomic studies may reveal its regulation in relation to adiposity, insulin resistance, or growth hormone signaling in youth [41].
- OLFM1: This is a brain-enriched protein that shows decreased levels in children with obesity [40].
- ANPEP (Aminopeptidase N): This points to alterations in the gut–metabolic axis or digestive enzyme regulation in obese children [43].
- FHL3 (Four and a Half LIM Domains 3): This may reflect impaired muscle development, lower lean mass, or altered muscle signaling in obesity [43].
- UMOD (Uromodulin): This suggests a kidney–metabolism connection; lower levels may indicate systemic inflammation or early renal stress [43].
- KIM1 (Kidney Injury Molecule-1): Elevated plasma levels correlate with decreased vagal heart rate variability (HRV), a marker of poor cardiovascular autonomic health. KIM1 is one of the three proteins (with IDUA and BOC) most consistently associated with at least three heart rate parameters, suggesting early cardiovascular stress in children with obesity [32].
- BOC (Brother of CDO): Elevated plasma BOC correlates positively with HRV. It is one of the top three proteins consistently linked across multiple HRV parameters, hinting at potential cardiometabolic benefits [40,42].
- IDUA (Alpha-L-iduronidase): Plasma IDUA levels are positively correlated with HRV—a higher IDUA means a better vagal tone. It is ranked among the key proteins associated with ≥3 HRV metrics, implying a potentially protective cardiovascular effect [42].
- GSTA1: Its elevation suggests enhanced antioxidant defense and a reduction in oxidative stress [40,41].
- MYH10: This points to improved muscle–adipocyte physiology and insulin signaling [41].
- CNDP1: Its changes correlate with BMI, but its role in obesity requires further investigation [41].
- CDH2 (Cadherin 2): A cell–cell adhesion molecule (N-cadherin), may reflect hepatic or adipose tissue remodeling, showing significant correlation with the severity of steatosis [45].
- CTSO (Catepsin O): This protease is involved in tissue remodeling, showing a significant correlation with the severity of steatosis [41,45].
- LILRA5 (Leukocyte Immunoglobulin-Like Receptor A5): This is an immune receptor on leukocytes; it is possibly linked to inflammation-driven liver pathology, showing a significant correlation with the severity of steatosis [27].
- ALPL (Alkaline Phosphatase): This is significantly upregulated in obese subjects. Elevated ALPL levels are correlated with cardiovascular risk factors, including systolic/diastolic blood pressure, mean arterial pressure, carotid intima–media thickness, and a trend toward higher fasting insulin, a consistent and novel neutrophil activation marker in obesity [34,84].

These biomarkers provide deeper insights into the complex interplay between metabolic processes and inflammation in the context of early-onset obesity. Their identification underscores the potential of proteomic analyses in uncovering novel targets for early detection and intervention strategies in pediatric obesity.

## 4. Discussion

Proteomic research in childhood obesity represents an incredibly promising and rapidly evolving area. Over the last fifteen years, significant technological innovations and the growing demand for personalized healthcare have led to the expansion of proteomic applications in pediatric obesity research.

In the context of obesity, proteomic analysis enables us to capture dynamic changes in metabolic and inflammatory pathways that may not be evident at the genomic or transcriptomic levels. Unlike the relatively stable genome, the proteome is highly dynamic and responsive to external factors, developmental stages, and disease processes, making it particularly valuable in pediatric research, where age-dependent biological variation is significant [7,14,15,19,20,24,26,36,41,49,76,77,81,85,86,87,88,89].

Serum proteomic analysis can identify novel biomarkers that complement standard clinical assays, enabling the early detection of metabolic impairments in obese children. This approach may enhance early intervention strategies and improve the management of childhood obesity. One of the most promising applications of proteomics in the context of pediatric obesity is its potential to stratify patients by metabolic risk and to predict disease progression or treatment response. As obesity is a heterogeneous condition, the ability to differentiate between metabolically healthy and unhealthy phenotypes in children is essential in early intervention and personalized care [18,24,35,90].

Our review highlights the way in which proteomic studies have emerged as a powerful approach to investigating the dynamic molecular dysregulations seen in obesity. Studies have consistently demonstrated that proteomic analyses can detect the distinct expression patterns of proteins involved in inflammation, oxidative stress, lipid metabolism, and hormonal regulation [89]. These findings can pave the way for individualized risk stratification, moving beyond traditional anthropometric metrics such as BMI to support the real-time monitoring of disease progression and treatment response [14,28,29,48]. These systems biology approaches enable clinicians to move beyond BMI, allowing for individualized risk profiling and the real-time monitoring of disease progression and treatment response [20,48,77,91].

Pediatric proteomic research faces challenges such as limited sample volumes, developmental variability, and ethical concerns. There is also the challenge of biological variability: children undergo constant physiological changes that affect protein levels, adding another layer of complexity [43,92].

Proteomic analyses have begun to uncover a range of candidate proteins that are differentially expressed in obese versus non-obese children. These biomarkers provide insights into the molecular mechanisms underpinning adipose tissue dysfunction, systemic inflammation, metabolic derangements, and early organ damage associated with obesity [7,11,18,22,37,45,46,53,59,62,81,93,94,95,96]. Proteomic studies in children commonly utilize non-invasive or minimally invasive biological samples such as plasma, serum, urine, and saliva. Each matrix offers distinct advantages and limitations. For example, plasma and serum are rich sources of circulating proteins involved in metabolic and immune processes but pose challenges due to the wide and dynamic range of protein concentrations. Urine and saliva, while more accessible, may contain a lower protein content and require more sensitive analytical techniques [38,40,42,97], and adipose tissue is less known. However, we could only find proteomic studies in adult cohorts. These findings suggest that proteomics may allow for the subclinical detection of metabolic impairment before overt obesity or comorbidities develop. The research below provides insights into the proteomic differences associated with metabolic health status in children with overweight or obesity, emphasizing the importance of early identification and intervention. Further large-scale studies are needed to confirm these findings.

Several longitudinal studies have examined how proteomic markers change in response to weight loss through diet and exercise. The normalization of APOA1 and a reduction in inflammatory proteins such as haptoglobin and S100A8/A9 have been observed following even modest reductions in body weight [15,41,98]. However, these findings are derived from relatively small cohorts, often lacking ethnic diversity, and the analytical platforms used vary substantially between studies. Without harmonized protocols and longitudinal validation, the clinical utility of APOA1, haptoglobin, and S100A8/A9 as surrogate markers of therapeutic efficacy in pediatric populations remains to be confirmed.

In addition to weight loss, dietary composition and physical activity have also been linked to distinct proteomic signatures. Studies have demonstrated that healthy dietary patterns are associated with proteomic profiles indicative of reduced risk for obesity-related complications. Proteins related to inflammation, lipid metabolism, and oxidative stress can be modulated by specific dietary components, which may support the development of tailored nutrition strategies for children at risk of metabolic disease [11].

Thrush’s study investigated why some obese (“diet-resistant”) individuals struggle to lose weight. Differences in muscle metabolism and plasma proteomic signatures pointed to intrinsic impairments in fat oxidation and energy expenditure. A study on six children with obesity undergoing a 30-day weight loss program identified changes in 43 proteins and 165 metabolites post-intervention, highlighting the molecular impact of weight loss [12].

Moreover, physical activity interventions have been shown to induce significant shifts in the proteome. Exercise-based randomized controlled trials in adolescents have identified changes in numerous immune-related proteins and inflammatory markers. In the study by Ramirez-Velez et al., they analyzed 65 immune-related proteins before and after a 6-month exercise intervention in adolescents, revealing changes in inflammatory markers [36]. Rodriguez-Ayllon’s RCT found that a 20-week exercise program significantly reduced MSR1 (macrophage scavenger receptor type I) levels in overweight or obese children, linking activity with brain health biomarkers [38]. Despite the observed reduction in MSR1 levels, these results require cautious interpretation, as current evidence is limited to single-center trials with modest sample sizes, and inter-assay variability may influence reproducibility across laboratories.

In terms of pharmacological treatment, Alshahrani et al. compared plasma protein expression among obese individuals, obese individuals with type 2 diabetes, and those treated with metformin. They identified 30 proteins with reliable sequence matches, including calbindin, apolipoprotein A-I, albumin, haptoglobin, annexin A3, and alpha-2-macroglobulin, involved in lipid transport, inflammation, oxidative stress, and cellular regulation. The study provides insights into how metformin modulates plasma protein profiles in obese diabetic patients, particularly across inflammation, oxidative stress, and metabolic pathways [48]. However, the cross-sectional nature of most studies and the confounding effects of concurrent lifestyle interventions limit the ability to attribute these proteomic changes solely to pharmacological treatment.

Large-scale studies employing advanced platforms such as SomaScan have expanded our understanding of the pediatric plasma proteome. The identification of hundreds of proteins associated with BMI and metabolic dysfunction supports the use of proteomic biomarkers as predictive tools in the early detection of comorbidities. The largest pediatric MS-based proteomic analysis to date, expanding our understanding of how biology and genetics shape the plasma proteome during growth, took place in the 2005 study by Niu et al. The study identified protein markers that can predict BMI, making them candidates for early diagnostic tools for metabolic risk in children [41,43]. Furthermore, combinations of proteomic biomarkers have demonstrated improved predictive power over traditional measures such as BMI or simple biochemical markers alone. Multiplex panels incorporating inflammatory, lipid, and hormonal proteins could facilitate risk stratification, helping clinicians to identify children who would benefit from intensified monitoring or early lifestyle interventions [63]. Although such multiplex panels show promise, differences in analytical sensitivity, the absence of standardized pediatric reference ranges, and population-specific variability still limit their readiness for routine clinical implementation.

Rothwell et al.’s paper [29] reports on a study on a group of overweight adolescent girls (13–17) and shows a specific proteomic signature in plasma associated with the HOMA value. It is clear that proteomic analysis of the plasma proteome in obese adolescents can potentially yield valuable information concerning the underlying changes in protein expression and metabolic changes that give rise to metabolic syndrome. Nevertheless, HOMA-related proteomic associations can be influenced by factors such as pubertal stage, acute illness, or nutritional status, which should be accounted for in future validation studies.

The final intervention treatment for severe pediatric obesity is bariatric surgery, which has emerged as an effective treatment for sustained weight loss and the resolution of comorbidities. Preliminary proteomic studies on adolescents undergoing sleeve gastrectomy or Roux-en-Y gastric bypass have identified dramatic shifts in circulating protein profiles within months post-surgery. These include the restoration of favorable lipid-related proteins (e.g., APOA1), reductions in pro-inflammatory markers, and improvements in oxidative stress proteins [77,91].

Oberbach et al. designed a study assessing six children with morbid obesity in relation to proteomic changes. The study demonstrates that LSG in children and adolescents leads to significant metabolic and proteomic alterations, reflecting improvements in lipid metabolism and reductions in obesity-related biomarkers. These changes suggest enhanced metabolic health and a decreased risk of obesity-related complications following surgery [31,62]. Still, these findings stem from very small cohorts, and replication in larger, multi-center pediatric surgical populations is necessary before such proteomic changes can be integrated into clinical monitoring protocols.

Some papers show a connection between obesity-related complications and changes in the proteome, such as obstructive sleep apnea, metabolic dysfunction-associated fatty liver disease, hepatic steatosis, kidney fibrosis and renal disease, and even asthma in the context of obesity [27,44,46,99,100,101,102,103].

Moving on to inflammation, we found several studies covering this subject; for example, Manell et al. employed the proximity extension assay (PEA) to quantify 113 inflammation-related proteins, identifying IL-33, IL-17C, and FGF-23 as potential biomarkers in adolescents with both asthma and obesity [104].

Similarly, Beglarian and colleagues analyzed a broad panel of 653 proteins, focusing on immune and inflammatory markers, cytokines, and metabolic and cardiovascular-related proteins, to explore their association with changes in bone mineral density and obesity in youth [45].

Rahman et al. applied a bead-based multiplex immunoassay to measure plasma levels of IGFBP1, IGFBP3, and IGFBP7 in a cohort of 420 adolescents aged 11–14 years, identifying IGFBP1 as a sensitive biomarker of obesity-related metabolic complications [55]. However, IGFBP1 concentrations can be influenced by non-obesity-related factors such as growth stage, acute inflammation, or nutritional status, which should be addressed in future studies.

In a distinct population, Sztolsztener et al. investigated apolipoprotein profiles in 58 childhood survivors of acute lymphoblastic leukemia (ALL), linking several apolipoproteins (APOA1, A2, C1, C3, H, J, and D) with obesity-related metabolic risk [105].

Importantly, the integration of proteomics into other omics technologies, including multi-omics approaches, could offer valuable insights into early diagnosis, personalized risk assessment, and therapeutic targeting [16,17,41,62,64,80,100,106,107].

For example, a meta-analysis incorporating genomics, metabolomics, and epigenomics could provide a comprehensive systems-level perspective on childhood obesity [108]. A study by Hellmuth et al. spanning multiple European pediatric cohorts revealed distinct amino acid and lipid metabolomic signatures in obese and insulin-resistant children [109]. Pan et al. identified ALPL as a marker of neutrophil activation associated with obesity [34], while Inoue et al. reported that overweight children exhibited altered lyso-PE lipid profiles, indicating early metabolic shifts before conventional lipid abnormalities became evident [74].

Furthermore, Concepcion et al. conducted the targeted metabolomic profiling of plasma and 24 h urine in 90 adolescents, stratified into three groups (obesity with T2D, obesity without T2D, and normal-weight controls). Using LC-MS/MS, they quantified 273 analytes and identified 22 urinary metabolites specifically associated with T2D. They found that urinary branched-chain amino acids (BCAAs) and their intermediates were more specific biomarkers for T2D and that plasma BCAAs were associated with obesity and insulin resistance, regardless of diabetes status [110]. While urinary BCAAs show promise, variability in dietary protein intake, hydration status, and sample handling can affect quantification, underscoring the need for standardized pre-analytical protocols [111].

Despite the promise shown, the translation of proteomic discoveries into clinical practice in pediatric obesity faces challenges. Current limitations include a lack of standardized protocols and methodologies, variability due to age, sex, and comorbidities, and longitudinal data. To overcome these barriers, future research should prioritize large-scale, multicenter cohort studies with long term follow-up. These studies are essential in validating candidate biomarkers, establishing pediatric reference ranges, and refining clinical risk stratification tools.

## 5. Conclusions

The rapidly advancing field of omics-based research, particularly proteomics, offers promising opportunities for identifying biomarkers of systemic metabolic disturbances in children with obesity. Across the reviewed literature, several candidates have emerged as particularly promising, including APOA1, haptoglobin, S100A8/A9, MSR1, IGFBP1, and BCAAs. These biomarkers hold potential for integration into clinical diagnostics, risk prediction, and the monitoring of therapeutic interventions.

While technical and interpretive challenges remain—especially in pediatric populations—the future of proteomics in childhood obesity is encouraging. Progress in mass spectrometry, bioinformatics, and multi-omics integration will enhance our ability to translate proteomic discoveries into meaningful clinical applications. Incorporating these insights into routine pediatric care could pave the way for a precision medicine framework, enabling more personalized, targeted, and effective treatment strategies.

In conclusion, the intersection of childhood obesity and proteomics research holds the potential to transform approaches to prevention, diagnosis, and therapy. However, it is essential that we overcome methodological barriers and bridge the gap between research and practice. Standardization and integration across omics platforms will be critical in unlocking the full potential of precision medicine for pediatric obesity and improving long-term outcomes for children with early-onset obesity worldwide.

## Figures and Tables

**Table 1:** Proteomic studies in pediatric obesity.

Study	Cohort	Method	Key Findings	Protein
Giraudi et al. (2011) [27]	59 obese adolescents (11–18 yo)	Proximity extension assay (PEA)	Focused on liver steatosis	CDH2, CTSO, and LILRA5
Galata et al. (2011) [28]	Prepubertal boys (10 normal, 10 overweight, 10 obese)	2-DE + MS	Changes in apolipoproteins highlight early dyslipidemia	APOA1, APOE, APOA4, CFB, CFH, CFI, HP, and CD5L
Rothwell et al. (2011) [29]	5 girls (13–17 yo), but the control group comprised adult females	Clontech antibody microarrays	Plasma proteomics can reflect metabolic dysfunction in obese youth	Identified plasma protein signatures (groups of proteins) that correlate with insulin resistance (e.g., hILP/XIAP, Nup888, Hap-1, Ki-67, TNFa, and ARPK1)
Gozal et al. (2011) [30]	120 children with overweight or obesity	2D-DIGE + mass spectrometry + Western blotting	Pediatric obstructive sleep apnea (OSA) biomarker discovery	UMOD, UCN3, ORM1, KLK1, and AZGP1
Oberbach et al. (2012) [31]	6 morbidly obese children and adolescents	Likely LC-MS/MS (not specified)	Shifts in lipid metabolism, insulin sensitivity, and oxidative stress after bariatric procedures	CLU, PEDF, RBP4, and PON1
Walker et al. (2014) [32]	42 children and adolescents aged between 5 and 18 yo	Two-dimensional electrophoresis (2-DE) and then mass spectrometry (MS) and immunoblotting	The proteomic approach highlights multimeric adiponectin as a potential molecular link between pediatric obesity and vitamin D deficiency. Vitamin D deficiency was associated with the downregulation of high-molecular-weight adiponectin, a protein linked to insulin sensitivity	Adiponectin
Martos-Moreno et al. (2014) [18]	22 obese prepubescents and 21 healthy prepubescents; 20 were obese before intervention and after weight reduction	Two-dimensional gel electrophoresis (2DE) + mass spectrometry (MS)	The study identified differential expression of proteins (231) suggesting early metabolic alterations in obesity	APOA1, Apo-J-clusterin, vitamin D binding, transthyretin, and haptoglobin
López-Villar et al. (2015) [33]	Review article on proteomic research tools and their applications in obesity and type 2 diabetes	Mass spectroscopy SRM (Selected Reaction Monitoring) or MRM (Multiple Reaction Monitoring) proteomic profiling of inflammatory pathways/bead-based targeted proteomics (e.g., Luminex, quantifying ~49 metabolic/inflammatory proteins) in serum	Obesity and diabetes linked to inflammatory protein expression	Complement C3, C5–C7, and lipid transport proteins
Pan et al. (2019) [34]	12 obese vs. 12 lean African American male adolescents (aged 14–20)	Label-free quantitative proteomics (likely LC–MS/MS)	Novel protein associated with obesity inflammation ALPL	ALPL
Zhen et al. (2021) [35]	60 (13–18 yo) Chinese normal weight/overweight/obese adolescents	TMT (Tandem Mass Tag)	Galectin-3BP associated with obesity and metabolic syndrome	Galectin3bp
Ramirez-Velez et al. (2022) [36]	95 adolescents aged 11–17 yo	A multiplex proteomic assay	IGFBP1 is a sensitive marker of obesity-related metabolic risk	BLC, Eotaxin, MCP-4, FGF-6, and PARC
Selvaraju et al. (2022) [37]	76 children (6–10 yo)	Multiplexed measurements	Saliva-based assays can detect key metabolic biomarkers	Fetuin-A, insulin, and adiponectin identified as non-invasive biomarkers
Rodriguez-Ayllon et al. (2023) [38]	81 overweight/obese children ~10 yo	Olink 92 protein panel (PEA)	Novel biomarkers influenced by lifestyle interventions. While the exercise program did not affect the selected candidate biomarkers, the identification of MSR1 as a responsive protein suggests potential avenues for future research into its role in health and disease	msr1
Pyle et al. (2024) [39]	Youth-onset T2D 374 baseline plasma samples; >90% had obesity	Multiprotein signature via mass spectrometry	Biomarkers identified for early diabetic kidney disease in obese children	SEM4A, DDC, C1RL1, and pyruvate carboxylase are linked to kidney risk
Olvera-Rojas et al. (2024) [40]	84 10-yo with overweight/obesity	Olink 92 protein panel	Neurological/metabolic proteins such as CD38 and LAIR2 distinguish metabolic health within obesity	CD38, CPM, EDA2R, IL12, JAMB, KYNU, LAYN, MSR1, SMOC2, and LAIR2
Liu et al. (2024) [41]	6 children (10–14 yo)	TMT and LC-MS	The study identified 43 differentially expressed proteins and 165 metabolites, highlighting pathways involved in lipid metabolism and inflammation	LCAT, GSTA1, PRCP, MYH10, and CNDP1
Plaza-Florido et al. (2024) [42]	44 (10-yo)	Olink 92 protein panel	Potential novel biomarkers linking cardiovascular-related plasma proteins to autonomic nervous system function in children with overweight or obesity	Eight proteins—KIM1, IgG Fc receptor II-b, IDUA, BOC, IL1RL2, TNFRSF11A, VSIG2, and transferrin (TF)
Niu et al. (2025) [43]	2147 children/adolescents (12-yo)	MS-DIA, high-throughput methods	Identified genetic determinants of proteome variation, BMI-related protein variation, pathway references	CRP, ACPS, SAA1, C3, CFH, CFI, ORM1, LBP, and PRG4
Díaz et al. (2025) [44]	Cord blood-derived exosomes collected at birth from 20 AGA (appropriate-for-gestational-age) and 20 SGA (small-for-gestational-age) infants	Label-free MS followed by bioinformatic pathway and protein–protein interaction (PPI) analyses	Exosome proteomics at birth can reveal protein signatures that predict metabolic outcomes such as insulin resistance and liver fat accumulation later in childhood	PCYOX1 and HSP90AA1 are predictive for liver fat at age 7
Beglarian et al. (2025) [45]	304 (8–13 yo) Hispanic children and 169 (17–22 yo) Hispanic adolescents	Olink 650 protein panel	44 inflammation-related proteins, e.g., early biomarkers for impaired bone health	PI3K-Akt pathways

**Table 2:** Proteomic biomarkers in childhood obesity. ↓ decrease; ↑ increase.

Biomarker	Source	Associated Complication	Proteomic Method	Notes
APOA1 (Apolipoprotein-A1)	Plasma, serum	Insulin resistance, lipid metabolism	2D-DIGE, LC-MS/MS	Reduced in obese and insulin-resistant children; reversible with weight loss
CLU (Apolipoprotein J, Clusterin)	Plasma, serum	Lipid transport, oxidative stress	2D electrophoresis, MS	Reduced in obese children
HP (Haptoglobin)	Plasma, serum	Inflammation, insulin resistance	2D-DIGE, MS	Low MW isoforms increased; correlates with IL-6, visfatin
CFB, CFH, CFI (Complement Factors B,H,I)	Plasma, serum	Immune activation, obesity	MALDI-TOF/MS analysis of 2-DE	Elevated in obese children
VDBP (Vitamin D Binding Protein)	Plasma	Vitamin D metabolism	LC-MS/MS	Reduced in obesity; linked with adiponectin pathway
HMW (Adiponectin)	Plasma	Vitamin D deficiency in obesity	2D-DIGE + MS + Western blot	↓ in vitamin D-deficient obese children; ↑ after 1 year of vitamin D supplementation
TTR (Transthyretin)	Plasma, serum	Thyroid hormone and retinol transport	LC-MS/MS	Decreased in obesity
PEDF	Adipose tissue	Insulin resistance, inflammation	Proteomic profiling	Induces insulin resistance and inflammation
RBP4	Plasma	Insulin resistance, endothelial dysfunction	2D-DIGE, MS	Elevated in obese children
PON1	Serum	Oxidative stress, lipid metabolism	Activity assays, MS	Altered activity in obesity
ALDH (Aldehyde dehydrogenase), ALB (Albumin), HSPB1, PDIA3	Plasma	Stress response, detoxification	LC-MS/MS	Altered expression in obesity
Lyso-PEs (16:0, 22:6)	Plasma	Lipid signaling	Lipidomics	Elevated in obese children
IGFBP1	Plasma	Growth factor regulation	Immunoassays, MS	Reduced in obesity
CRP	Plasma	Chronic inflammation	Immunoassays	Correlates with BMI in obesity
S100-A8, S100-A9, calprotectin	Plasma	Inflammation	Proteomics	Overexpressed in obesity
PTX3	Plasma	Cardiovascular inflammation	TMT-labeled LC-MS/MS and ELISA	Early marker for CV risk
Galectin 3BP	Serum	Obesity, metabolic syndrome	TMT-labeled LC-MS/MS and ELISA	Marker for metabolic syndrome risk
PCYOX1, HSP90AA1	Plasma	Liver fat prediction	Targeted proteomics	Predictive for liver steatosis
RAS	Plasma, Cellular	Insulin signaling	Antibody microarrays: Clontech	Altered in obesity
PKC-η	Plasma, Cellular	Inflammation	Clontech	Upregulated in inflammation
PDK1	Plasma, Cellular	Insulin/PI3K pathway	Antibody microarrays	Altered expression
STAT3	Plasma, Cellular	Inflammation, metabolism	Antibody microarrays	Activated in obesity
p38 MAPK	Plasma, Cellular	Stress response	Antibody microarrays	Increased in metabolic stress
AHSG (Fetuin-A)	Plasma, saliva	Linked to insulin resistance	2D-MS differential spots	Correlates with insulin resistance and adiposity
CD38	Plasma	Regulation of cell growth, insulin secretion	LC-MS/MS	Altered expression in cardiovascular and neurological diseases and aging
MSR1	Plasma	Immune response, lipid transport, inflammation metabolism	LC-MS/MS, Olink	Atherosclerosis—positively associated with TG levels and in clearing infectious agents
MANF	Plasma	Neuron projection development	LC-MS/MS, Olink	Neurological disease
LAIR2	Plasma	Collagen binding	LC-MS/MS, Olink	Immunological response in obese
OLFM1	Plasma	Neurological system	Olink	Decreased in obese children; may be involved in neurodevelopment and synaptic plasticity
ANPEP	Plasma	Digestive system and immune	Olink	Regulates peptide digestion and immune signaling
FHL3	Plasma	Skeletal system	Olink	Muscle development, differentiation, and function
UMOD	Plasma	Urological system	Olink	Anti-inflammatory; protects renal function
KIM	Plasma, urine	Urological system	Olink	Elevation in obese youth indicates subclinical kidney stress
BOC	Plasma, serum	Cardiometabolic system	Olink	Early signs of metabolic and organ-specific stress
IDUA	Serum	Cardiovascular system	Olink	Implying a potentially protective cardiovascular effect
CDH2	Serum	Cardiometabolic system	Olink PEA	Liver steatosis
CTSO	Serum	Cardiometabolic system	Olink PEA	Liver steatosis
LILRA5	Serum	Cardiometabolic system	Olink PEA	Liver steatosis
SEM4A, PSB3, DDC, C1RL1, T132A	Plasma	Kidney disease	SomaScan v.7K	Multiprotein plasma signatures that strongly predict early DKD outcomes in youth-onset T2D—far outperforming clinical markers alone
ALPL	Purified neutrophils	Cardiovascular system	Quantitative, label-free mass spectrometry proteomics performed on purified neutrophils	ALPL is consistently elevated in obese neutrophils and correlates with CVD risk markers
NRP2	Plasma	Metabolic health status	Olink	Decrease in metabolic health in children with obesity versus children with metabolic syndrome and obesity
SSP1 (Osteopontin)	Plasma	Bone mineral density, cardiovascular system	Olink	Increase in bone mineral density gains in obese children
MFAP5	Plasma	BMD dynamics	Olink (inflammatory and cardiometabolic panel)	Biphasic: ↑ at baseline, ↓ longitudinally
GSTA1	Serum	BMI and oxidative stress after weight loss	TMT-labeled LC-MS/MS	Decrease after weight loss
MYH10	Serum	Obesity-related adipogenesis	TMT-labeled LC-MS/MS	Decrease after weight loss, regulates adipocyte GLUT4 activity
CNDP1	Serum	Obesity and metabolic response	TMT-labeled LC-MS/MS	Increase after weight loss

## Supplementary Materials

The following supporting information can be downloaded at: https://www.mdpi.com/article/10.3390/ijms26178522/s1.

## Abbreviations

The following abbreviations are used in this manuscript:

2D-DIGE	2-dimensional Differential Gel Electrophoresis
BMI	Body Mass Index
DIA	Data-Independent Acquisition
ELISA	Enzyme-Linked ImmunoSorbent Assay
LC-MS/MS	Liquid Chromatography–Tandem Mass Spectrometry
LSG	Laparoscopic Sleeve Gastrectomy
MALDI-TOF	Matrix-Assisted Laser Desorption/Ionization Time-Of-Flight
MHO	Metabolically Healthy Obese
MRM	Multiple Reaction Monitoring
MS	Mass Spectrometry
MUO	Metabolically Unhealthy Obese
SRM	Selected Reaction Monitoring
T2D	Type 2 Diabetes
TMT	Tandem Mass Tagging
PEA	Proximity Extension Assay
PPI	Protein–Protein Interaction
RCT	Randomized Controlled Trial
