# Supplementary material for: Proteomic Insights into Childhood Obesity: A Systematic Review of Protein Biomarkers and Advances
Source: Int J Mol Sci. 2025 Sep 2;26(17):8522. doi: 10.3390/ijms26178522 (PMC12429325; doi:10.3390/ijms26178522)
Supplement: Supplementary file 1 [file ijms-26-08522-s001.zip › Suppl File 1 Proteomic Technologies.docx]

**Proteomic Technologies**

Several proteomic platforms have been employed in the study of childhood obesity, including:

• Two-Dimensional Gel Electrophoresis (2D-GE): An early method that separates proteins based on isoelectric point and molecular weight. Although still used, it has largely been supplanted by more advanced techniques due to limited sensitivity and throughput.

• Liquid Chromatography–Tandem Mass Spectrometry (LC-MS/MS): Now considered the gold standard in proteomic research. It offers high sensitivity, accuracy, and the ability to identify and quantify thousands of proteins from complex biological samples – and this is the most common method in found papers.

**Mass Spectrometry (MS)**
Mass spectrometry is an analytical technique that identifies and quantifies molecules based on their mass and electrical charge. In proteomics, MS is used to separate proteins into smaller fragments (peptides), measure their precise masses, and compare these measurements to protein databases to determine their identity and abundance. The process typically involves:

1. **Sample preparation** – proteins are extracted from the biological sample and broken down into peptides.
2. **Ionization**– peptides are converted into charged particles.
3. **Mass analysis** – the instrument measures how quickly these ions travel, which depends on their mass and charge.
4. **Data analysis** – the mass patterns are matched to known protein sequences to identify and quantify proteins.

This approach allows researchers to detect thousands of proteins simultaneously, even in very small or complex samples, and to capture subtle changes in the proteome that may be linked to disease.

• Isobaric Tagging (e.g., iTRAQ, TMT): These chemical labeling methods allow for multiplexed quantification of proteins across multiple samples in a single LC-MS/MS run, increasing throughput and reducing batch effects.

• Label-Free Quantification (LFQ): An increasingly popular alternative that eliminates the need for chemical labeling while maintaining high sensitivity and reproducibility.

• Targeted Proteomics (e.g., Selected Reaction Monitoring - SRM): Often used to validate findings from discovery-phase studies, these methods allow for precise quantification of selected proteins of interest.

The PEA (Proximity Extension Assay) is a highly sensitive method for measuring proteins in biological samples. It uses pairs of antibodies that specifically recognize and bind to the target protein. Each antibody is linked to a short piece of DNA.
When both antibodies bind to the same protein, their attached DNA strands are brought close together (“in proximity”) and can be joined to form a new piece of DNA. This newly formed DNA sequence serves as a unique identifier for the protein and can be quantified using real-time polymerase chain reaction (PCR).

The key advantages of PEA are:

- It requires only very small sample volumes (a few microliters), making it suitable for pediatric studies.
- It can measure many proteins at once (multiplexing) with high sensitivity and specificity.
- It minimizes cross-reactivity compared to traditional antibody-based assays.

By combining antibody specificity with the precision of DNA quantification, PEA enables accurate detection of low-abundance proteins in complex biological samples.

The technique was developed by Olink Proteomics.

**Glossary**

| Term | Definition |
| --- | --- |
| Proteomics | The large-scale study of the complete set of proteins (proteome) in a biological sample, including their structure, function, and changes under different conditions. |
| Biomarker | A measurable indicator of a biological state, condition, or disease, used for diagnosis, prognosis, or monitoring treatment response. |
| Mass Spectrometry (MS) | An analytical technique used to measure the mass-to-charge ratio of ions, allowing precise identification and quantification of proteins and other molecules in complex samples. |
| Proximity Extension Assay (PEA) | A high-sensitivity method for detecting proteins using pairs of antibodies linked to DNA strands, which form a unique DNA template when bound to the target protein, enabling quantification via PCR. |
| Branched-Chain Amino Acids (BCAAs) | Essential amino acids (leucine, isoleucine, and valine) involved in protein synthesis and energy production; elevated levels in biofluids have been associated with obesity and insulin resistance. |
| Apolipoprotein A-I (ApoA1) | The main protein component of high-density lipoprotein (HDL), involved in lipid transport and cardiovascular protection. |
| S100A8/A9 (Calprotectin) | A protein complex associated with inflammation, immune response, and oxidative stress; elevated levels are linked to obesity-related inflammation. |
| MSR1 (Macrophage Scavenger Receptor Type I) | A membrane receptor involved in lipid metabolism, immune regulation, and inflammatory processes. |
| IGFBP-1 (Insulin-like Growth Factor Binding Protein 1) | A protein that binds insulin-like growth factors, regulating their activity; levels are influenced by nutritional status and metabolic health. |
| Multi-omics | An integrated approach that combines different 'omics' technologies (e.g., genomics, proteomics, metabolomics) to provide a comprehensive view of biological systems. |
