# Supplementary material for: Proteomic Insights into Childhood Obesity: A Systematic Review of Protein Biomarkers and Advances
Source: Int J Mol Sci. 2025 Sep 2;26(17):8522. doi: 10.3390/ijms26178522 (PMC12429325; doi:10.3390/ijms26178522)
Supplement: Supplementary file 1 [file ijms-26-08522-s001.zip › suppl file 2 proteomic search algorithm.docx]

**Supplementary File Proteomic Article Search Algorithm**

**Pubmed** (("child"[MeSH Terms] OR "children"[All Fields] OR "pediatric"[All Fields] OR "adolescent"[MeSH Terms] OR "adolescents"[All Fields]) OR "childhood"[All Fields]

AND

("obesity"[MeSH Terms] OR "overweight"[All Fields] OR "obese"[All Fields])

AND

("proteomics"[MeSH Terms] OR "proteomic"[All Fields] OR "protein expression"[All Fields] OR "mass spectrometry"[MeSH Terms] OR "2D gel electrophoresis"[All Fields] OR "protein profiling"[All Fields] OR "protein biomarker"[All Fields])

AND

("biomarkers"[MeSH Terms] OR "biomarker"[All Fields] OR "differential protein expression"[All Fields])

**Scopus**: (TITLE-ABS-KEY(child* OR adolescen* OR pediatric) AND

TITLE-ABS-KEY(obesity OR overweight OR obese) AND

TITLE-ABS-KEY(proteomics OR "protein expression" OR "mass spectrometry" OR "protein profiling") AND

TITLE-ABS-KEY(biomarker*))

AND (LIMIT-TO(DOCTYPE, "ar"))

AND (LIMIT-TO(LANGUAGE, "English"))

AND (LIMIT-TO(SUBJAREA, "MEDI") OR LIMIT-TO(SUBJAREA, "BIOC"))

**Web of Science:** TS=(child* OR adolescen* OR pediatric) AND

TS=(obesity OR overweight OR obese) AND

TS=(proteomics OR "protein expression" OR "mass spectrometry" OR "protein profiling") AND

TS=(biomarker*)
